# Real-World Outcomes of Anti-VEGF Treatment for Neovascular Age-Related Macular Degeneration in Turkey: A Multicenter Retrospective Study, Bosphorus Retina Study Group Report No: 1

**DOI:** 10.4274/tjo.31697

**Published:** 2018-10-31

**Authors:** Abdullah Özkaya, Levent Karabaş, Cengiz Alagöz, Zeynep Alkın, Özgür Artunay, Selim Bölükbaşı, Gökhan Demir, Mehmet Demir, Ali Demircan, Burak Erden, Gürkan Erdoğan, Mehmet Erdoğan, Erdem Eriş, Havva Kaldırım, İsmail Umut Onur, Özen Osmanbaşoğlu, Sezin Özdoğan Erkul, Mine Öztürk, İrfan Perente, Kübra Sarıcı, Nihat Sayın, Dilek Yaşa, İhsan Yılmaz, Zeynep Yılmazabdurrahmanoğlu

**Affiliations:** ¤ Bosphorus Retina Study Group; 1 Beyoğlu Eye Training and Research Hospital, Ophthalmology Clinic, İstanbul, Turkey; 2 Kocaeli University Faculty of Medicine, Department of Ophthalmology, Kocaeli, Turkey; 3 Okmeydanı Training and Research Hospital, Ophthalmology Clinic, İstanbul, Turkey; 4 Şişli Etfal Training and Research Hospital, Ophthalmology Clinic, İstanbul, Turkey; 5 Kanuni Sultan Süleyman Training and Research Hospital, Ophthalmology Clinic, İstanbul, Turkey; 6 Bağcılar Training and Research Hospital, Ophthalmology Clinic, İstanbul, Turkey; 7 Bakırköy Training and Research Hospital, Ophthalmology Clinic, İstanbul, Turkey; 8 İstanbul Training and Research Hospital, Ophthalmology Clinic, İstanbul, Turkey; 9 Haseki Training and Research Hospital, Ophthalmology Clinic, İstanbul, Turkey

**Keywords:** Age-related macular degeneration, anti-vascular endothelial growth factor, treatment

## Abstract

**Objectives:**

To evaluate the real-world outcomes of intravitreal anti-vascular endothelial growth factor (anti-VEGF) treatment in neovascular age-related macular degeneration (nAMD) patients.

**Materials and Methods:**

Multicenter, retrospective, interventional, non-comparative study. The records of nAMD patients treated with an anti-VEGF agent on a pro re nata treatment regimen basis between January 2013 and December 2015 were reviewed. The patients who completed a follow-up period of 12 months were included. Primary outcome measures of this study were the visit and injection numbers during the first year.

**Results:**

Eight hundred eighty eyes of 783 patients met the inclusion criteria for the study. Mean number of visits at month 12 was 6.9±2.5 (range: 1-15). Mean number of injections at month 12 was 4.1±1.9 (range: 1-11). Mean visual acuity at baseline and months 3, 6, and 12 was 0.90±0.63 LogMAR (range: 0.0-3.0), 0.79±0.57 LogMAR (range: 0.0-3.0), 0.76±0.57 LogMAR (range: 0.0-3.0), and 0.79±0.59 LogMAR (range: 0.0-3.0), respectively. Mean central retinal thickness at baseline and months 6 and 12 was 395±153 μm (range: 91-1582), 330±115 μm (range: 99-975), and 332±114 μm (range: 106-1191), respectively.

**Conclusion:**

The numbers of visits and injections were much lower than ideal and were insufficient with the pro re nata treatment regimen.

## Introduction

Before the introduction of anti-vascular endothelial growth factors (anti-VEGF), the goal of treatment in neovascular age-related macular degeneration (nAMD) was only to prevent vision loss.^1,2,3,4,5,6,7^ The first labeled intravitreal drug for the treatment of nAMD was pegaptanib, then off-label drug bevacizumab and approved drugs aflibercept and ranibizumab have led us to prevention of vision loss in most of the nAMD patients and vision gain in one third of them.^4,5,6,7,8^ Several treatment regimens were evaluated in randomized controlled trials for each drug. Fixed monthly, pro re nata (PRN) with or without an initial 3 monthly loading doses, fixed bimonthly injection after the first 3 monthly loading doses, and “treat and extend” were some of the described treatment regimens.^5,6,7,8,9,10^ The monthly and PRN regimens were the earliest described.^3,5,6,7^ After the PrONTO study by Lalwani et al.^7^, the PRN regimen became popular worldwide, including in Turkey.^8^ Numerous studies were conducted to reevaluate the outcomes of this treatment regimen.^6,8,11,12^ Physicians liked the idea of seeing the patients every month and injecting when required, because the PRN regimen seemed to have the advantage of individualized dosing.^8^ However, most of the subsequently published real-world practice studies revealed that it was not possible to obey the strict follow-up and retreatment criteria of randomized controlled trials in daily practice.^10,11,13,14,15,16,17,18,19,20^ Most of these studies showed that the PRN regimen resulted in less frequent patient monitoring and injections. Several single-center and multicenter national studies were conducted to evaluate this phenomenon.^13,14,15,16,17,18,19,20^ Therefore, we conducted this multicenter study to assess the real-world outcomes of intravitreal anti-VEGF treatment in nAMD patients in 9 tertiary centers, all of which are located in or near İstanbul, the most populated city in Turkey, and we believe may reflect the general trends of treatment regimens in Turkey.

## Materials and Methods

This was a retrospective, interventional, non-comparative real-life experience study conducted in 9 tertiary centers in Turkey. The records of nAMD patients who were treated with an anti-VEGF agent using a PRN treatment regimen between January 2013 and December 2015 were reviewed. Written informed consent was obtained from all patients before the treatment and the study adhered to the tenets of the Declaration of Helsinki. Ethical board approval was obtained from Kocaeli University Faculty of Medicine.

Patients who met the following criteria were included in the study: were ≥50 years of age, were diagnosed with nAMD, and had a minimum follow-up time of 12 months. Patients who had retinal disease other than nAMD (e.g., diabetic retinopathy, retinal vein occlusion) and those diagnosed with polypoidal choroidal vasculopathy or retinal angiomatous proliferation during follow-up were not included.

Data collected from the patients included age, gender, lens status, the drug used, whether they were treatment-naïve or -experienced, whether they received a loading dose of 3 injections, the period over which the 3 loading doses were administered, BCVA and central retinal thickness (CRT) at baseline and months 3, 6, 9, and 12 as well as number of visits and injections given during the first year.

All patients underwent a standardized examination including measurement of BCVA via the Early Treatment Diabetic Retinopathy Study (ETDRS) chart or a projection chart at 4 meters, slit-lamp biomicroscopy and fundus examination, and measurement of intraocular pressure via applanation tonometry. Fundus photography, fluorescein angiography (FA), and optical coherence tomography (OCT) imaging were performed before treatment. As this was a multicenter study, different brands of FA and OCT devices were used to assess the patients. All examinations were planned to be repeated monthly, except FA. FA was repeated only when the cause of visual acuity deterioration could not be clarified with clinical examination and other imaging methods. Optical coherence tomography was used for detecting subretinal fluid and measurement of CRT. CRT, defined as the mean thickness of the neurosensory retina in the central 1 mm diameter area, was computed using OCT mapping software generated by the device. 

All injections were performed under sterile conditions in an operating room or an outpatient operating room (clean room). Topical anesthesia and 10% povidone-iodine (Betadine; Purdue Pharma, Stamford, CT, USA) were applied to the lids and lashes, and 5% povidone-iodine was administered to the conjunctival sac. Intravitreal bevacizumab 1.25 mg/0.1 mL, ranibizumab 0.5 mL/0.1 mL, or aflibercept 2 mg/0.1 mL was injected through the pars plana 3.5-4 mm posterior to the limbus with a 30-gauge needle. After the injection, an ophthalmic solution of 0.5% moxifloxacin (Vigamox; Alcon Laboratories, Inc., Fort Worth, Texas, USA) was administered 5 times a day for 1 week. Patients were then instructed to consult the hospital if they experienced decreased vision, eye pain, or any new symptoms.

Some of the patients initially received the three monthly loading doses of anti-VEGF, while others did not. The decision to give a loading dose was not made according to strict criteria but was based on the physicians’ preference. The patients were planned to be called for monthly visits. A single injection of a first preferred anti-VEGF agent was repeated when visual acuity decreased by one or more lines from the last visit or in the presence of newly developed macular hemorrhage, evidence of subretinal fluid, or persistent intraretinal fluid on OCT.

Primary outcome measures of this study included the numbers of visits and injections during the first year. Secondary outcome measures were change in BCVA and CRT from baseline to month 12.

### Statistical Analysis

Visual acuity was converted from decimals to the logarithm of the minimum angle of resolution (LogMAR) for statistical analysis. Categorical variables were presented as numbers and percentages, while numerical variables were expressed as mean and standard deviation. The data were assessed for normality using the Kolmogorov-Smirnov test. As the distribution of the data was found to be normal, changes in BCVA and CRT values between baseline and the other time points were assessed with repeated measures test. Categorical variables were compared using chi-square test. Statistical analyses were performed using SPSS (Version 21.0, SPSS Inc., Chicago, IL, USA). A p value <0.05 was considered statistically significant.

## Results

Eight hundred eighty eyes of 783 patients met the inclusion criteria for the study. The mean age was 73.2±8.8 years (range 50-94 years); 345 patients (44.0%) were women and 438 (56.0%) were men. One hundred thirty-eight eyes (15.7%) had been treated before, while 742 eyes (84.3%) were treatment-naïve. Thirty-six eyes (4.1%) received intravitreal bevacizumab, 222 eyes (25.2%) received intravitreal aflibercept, and 622 eyes (70.7%) received intravitreal ranibizumab as the initial treatment. The general characteristics of the patients are summarized in Table 1.

Mean number of visits at month 12 was 6.9±2.5 (range: 1-15). Mean number of injections at month 12 was 4.1±1.9 (range: 1-11). Two hundred eighteen eyes (24.8%) did not receive a loading dose of 3 consecutive monthly injections, whereas the other 662 eyes (75.2%) did. The mean duration for giving the loading dose of 3 injections was 83±22 days (range 56-150 days) in the subgroup of patients who received the loading doses.

**Figure 1:**
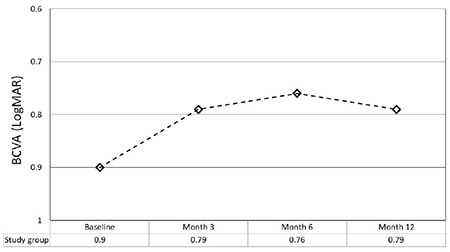
The change in mean best corrected visual acuity at different time points BCVA: Best corrected visual acuity

Mean BCVA at baseline and months 3, 6, and 12 was 0.90±0.63 LogMAR (range: 0.0-3.0), 0.79±0.57 LogMAR (range: 0.0-3.0), 0.76±0.57 LogMAR (range: 0.0-3.0), and 0.79±0.59 LogMAR (range: 0.0-3.0), respectively (Figure 1) (p<0.0001 for all). As this was not principally a study of effectiveness, we did not use visual acuity cut-off values while including the patients. However, we calculated the rate of the eyes which were stable, or lost ≥3 lines of vision at month 12 in the subgroup of eyes which had a BCVA between 1.3 and 0.3 LogMAR. There were 580 eyes in this subgroup and 175 (30.2%) of them showed ≥3 lines of gain in vision, 336 (57.9%) showed stable vision (stable, or <3 lines of visual gain, or <3 lines of visual loss), and 69 (11.9%) showed ≥3 lines of loss in vision.

**Figure 2:**
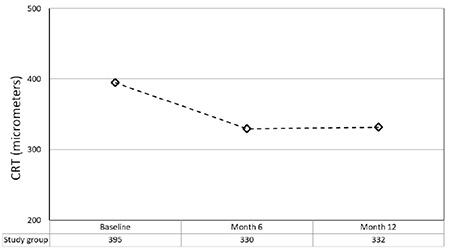
The change in mean central retinal thickness at different time points CRT: Central retinal thickness

Mean CRT values at baseline and months 6 and 12 were 395±153 µm (range: 91-1582), 330±115 µm (range: 99-975), and 332±114 µm (range: 106-1191), respectively (p<0.0001 for month 6 and 12) (Figure 2).

All complications were limited to subconjunctival hemorrhage, punctate epitheliopathy, and mild anterior chamber reaction. No endophthalmitis was detected in any of the eyes during the study period.

## Discussion

In the initial report of this multicenter study, we evaluated the real-world outcomes of intravitreal anti-VEGF treatment in nAMD patients, with the main focus on visit and injection numbers in the first year of treatment. All of the physicians who participated in this study reviewed the medical records of their patients and the data of 880 eyes of 783 patients were analyzed. The mean visit number was found to be 6.9 and injection number was 4.1. In the PrONTO study, the first year injection number was reported to be 5.6.^7^ However, time-domain OCT was used at the time that study was conducted, and it was later shown that time-domain OCT devices could not detect anatomical disease activity in at least one-third of the patients when compared with spectral-domain OCT devices.^21^ In other major prospective studies, the mean injection number required to treat nAMD during the first year was found to be 7-9 injections.^6,8,9,12^ However, this number was reported as low as 3-4 in most of the real-world practice studies.^13,14,15,16,17,18,19,20^ In addition, 12-13 visits are necessary in an ideal PRN treatment follow-up protocol.^6,7^ The mean visit number was 6.9 in our study. In several previous real-world studies the mean visit number was found to be between 6 and 12.^13,14,15,16,17,18,19,20^

The importance of giving the first 3 loading doses at the beginning of treatment for nAMD was documented in a previous study.^17^ The patients who received the first 3 loading doses demonstrated better visual outcomes than the patients who did not. The gain in visual acuity was 6 letters higher in the group of patients who received the loading dose than the patients who did not.^17^ We also evaluated our data in this regard and 75.2% of the included patients received the first 3 loading injections, whereas 24.8% of them did not. The time period for giving the 3 loading doses varied between 56 and 150 days, with a mean of 83 days. This duration should be 60 days in an ideal scenario.^7^

Bevacizumab was preferred as the first-line treatment in 4.1% of the eyes, whereas aflibercept was preferred in 25.2% and ranibizumab was preferred in 70.7%. As this study was a retrospective and non-randomized study, the drug choice seemed to be made upon the physicians’ preferences. Being an off-label drug, bevacizumab was used least frequently. Ranibizumab was the most frequently preferred drug, probably because it was the older of the two approved drugs. 

As the primary objective was to assess and discuss follow-up visit and injection numbers, we did not analyze visual and anatomical outcomes deeply in this report. The mean visual acuity was found to be improved from 0.90 LogMAR to 0.79 at month 12 and the mean CRT was reduced from 395 µm to 332 µm. 

In most real-world studies, visit and injection numbers were determined to be very far from the ideal.^13,14,15,16,17,18,19,20^ This may be secondary to heavy patient load, visit and injection scheduling problems, and patient compliance. Therefore, we might suppose that the PRN treatment regimen may not be suitable for the treatment of nAMD in daily practice according to our results and most of the other studies results. Although the mean visual acuity was found to be increased by 1.1 lines in our study, this report was not designed as an effectiveness study and patients with very low visual acuity were included, which might cause this phenomenon. In randomized controlled studies in which the follow-up and treatment criteria were strictly obeyed, fixed monthly injections of ranibizumab or bevacizumab, fixed bimonthly injections of aflibercept after 3 loading injections, PRN treatment regimens, and more flexible treatment regimens such as treat-and-extend have resulted in visual gains of 5-12 ETDRS letters after 12 months of follow-up.^3,5,6,7,8,9,12^ In the MARINA, ANCHOR, and CATT studies, monthly ranibizumab treatment regimen resulted in up to 11 letters of visual increase at month 12.^3,5,6^ The PRN regimen was also as effective as monthly treatment regimens according to CATT and IVAN study treatment arms.^6,12^ In addition to these treatment regimens, Wykoff et al.^22^ reported that ranibizumab provided 10.5 letters of visual increase at month 12 of a treat-and-extend regimen.

Aflibercept has also been evaluated with several treatment regimens.^8,23,24^ In the VIEW studies, monthly and bimonthly aflibercept treatment after 3 loading doses resulted in 8-9 letters of visual increase at month 12.^8^ Yamamoto et al.^25^ evaluated the efficacy of treat-and-extend regimen with aflibercept in nAMD and demonstrated 1.5 lines of visual increase at month 12. In all of these and other randomized controlled studies, the mean injection number during the first year was reported as at least 8.^6,8,12,23,24,25^ On the other hand, reaching this injection number seems nearly impossible with the PRN treatment regimen in real life.^13,14,15,16,17,18,19,20^ Most of the retrospective real-life studies reported the injection number as 3-4 during the first year.^13,14,15,16,17,18,19,20^ In a multicenter study, the mean injection number at month 12 was reported between 4.3 and 5.7 in patients from different countries.^13^ Two consecutive studies from France regarding real-life treatment of nAMD on a PRN regimen evaluated the results in two different time periods.^15^ The authors compared the outcomes in the second study.^15^ The LUMIERE study consisted of nAMD patients who were treated between 2006 and 2009 and the following TWIN study included patients who were treated between 2010 and 2011.^15,26^ They concluded that although improvements were made in key parameters, the mean injection number was around 5.5 at month 12. In addition, they pointed out the importance of regular postinduction monitoring (after 3 loading doses) and reported that it was the most important determinant of successful treatment.^15,26^ In a multinational real-life study by Holz et al.,^14^ patients from Canada, France, Germany, Ireland, Italy, the Netherlands, United Kingdom, and Venezuela were assessed. The mean number of injections was reported to be 5.0 at month 12 and the mean visual change was 2.4 letters. In conjunction with this study, several national reports were published by using national patient data. In a German real-life study by Ziemssen et al.,^16^ the mean number of anti-VEGF injections at month 12 was found to be 4.3, along with the 1.1 letters of visual increase. Among the countries involved the AURA study, the greatest mean injection number was reported from England.^17^ The mean number of injections at month 12 was 5.8 and the mean change in visual acuity was 6.0 letters. In other retrospective real-life studies, the mean injection number at month 12 was reported as 5.7 by Kataja et al.,^18^ 3.8 by Silva et al.,^19^ and between 3.7 and 4.9 by Jain et al.^20^ Nearly all of the real-life studies demonstrated that it was not possible to perform the proper number of visits and injections. After proving this fact, the performance of other treatment regimens was evaluated or compared with PRN regimen in new studies.^9,10,23^ Ozturk et al.^23^ retrospectively evaluated the outcomes of fixed bimonthly injection of aflibercept. They reported that 50% of the patients received the 8 obligatory injections during 12 months, and only 2 of the 42 patients were reported to receive 5 injections, which was the minimum injection number among the study patients. In another study from the United States by Lotery et al.,^24^ the mean numbers of ranibizumab and aflibercept injections were reported as 6.7 and 7.0, respectively.

Two interesting studies compared the difference between the PRN and treat-and-extend regimens in nAMD.^9,10^ In the TERRA study from the United Kingdom, the authors compared the mean injection number of patients previously treated PRN and then switched to treat-and-extend regimens.^9^ Interestingly, the mean number of injections during a 12-month follow-up on the PRN regimen was 4.7, and increased to 8.9 after switching to the treat-and-extend regimen. Johnston et al.^10^ conducted a real-life study based on the different treatment tendencies in Australia and the United Kingdom. They used Australia to analyze the treat-and-extend regimen and the United Kingdom for PRN. The mean injection numbers at month 12 were reported to be 9.2 in the treat-and-extend group and 6.0 in the PRN group.

### Study Limitations

The present study has several limitations. We did not evaluate the visual and anatomical outcomes of the study in detail in this initial report of our study group. We are planning a deeper assessment of these outcomes in future reports. However, this is an important national study in terms of the demographics and injection characteristics, which is a major strength.

## Conclusion

In conclusion, as proven by several multi- or single-center studies from different countries, it is very difficult to obey the strict follow-up and re-treatment criteria of the PRN regimen in nAMD patients.^13,14,15,16,17,18,19,20^ This was the first multicenter study from Turkey to demonstrate this phenomenon. The number of patients included was satisfactory for a multicenter study conducted in a country between Europe and the Middle East to show the treatment tendencies in nAMD. The number of visits and injections were far from ideal with the PRN treatment regimen. It is likely that all of the centers included will have to organize their clinical approach to the treatment of nAMD, try to perform more frequent injections and visits, or switch to another treatment regimen such as treat-and-extend or fixed regimens.

## Figures and Tables

**Table 1:**
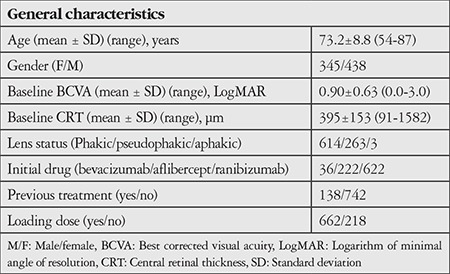
General characteristics of patients
